# Design of a yeast-based bio-interface on an optical fiber SPR platform toward drug adsorption monitoring

**DOI:** 10.3762/bjnano.17.89

**Published:** 2026-09-22

**Authors:** Natsumi Kitaya, Daisuke Yamanaka, Kenji Morita, Ryota Naito, Hinako Suhara, Kento Suzuki, Hizuru Nakajima, Yukiko Moriiwa, Kazuhiro Morioka, Akio Yanagida, Atsushi Shoji

**Affiliations:** 1 Laboratory for Bioanalytical Chemistry, School of Pharmacy, Tokyo University of Pharmacy and Life Sciences, Hachioji, Tokyo 192-0392, Japan; 2 Laboratory for Immunopharmacology of Microbial Products, School of Pharmacy, Tokyo University of Pharmacy and Life Sciences, Hachioji, Tokyo 192-0392, Japan; 3 Department of Applied Chemistry for Environment, Graduate School of Urban Environmental Sciences, Tokyo Metropolitan University, 1-1 Minami-osawa, Hachioji, Tokyo, 192-0397, Japan

**Keywords:** bio-interface, biosorption, optical fiber surface plasmon resonance, yeast

## Abstract

Yeast biomass, *Saccharomyces cerevisiae*, has been widely studied for removing heavy metals and organic pollutants, including pharmaceuticals. However, conventional batch methods provide limited insight into time-resolved adsorption and interfacial molecular interactions. We developed an optical fiber-based surface plasmon resonance (SPR) sensor incorporating a yeast-derived bio-interface. A film formation monitoring method based on transmitted light absorbance during electroless Ni plating enabled reproducible fabrication of gold nanofilms. Yeast cells were uniformly immobilized via a spray-based method to construct the bio-interface. Using this sensor, real-time adsorption of pharmaceuticals onto yeast cells was evaluated: Doxorubicin showed a concentration-dependent SPR response, whereas ampicillin showed no significant change in SPR response. The optical fiber configuration allows for direct immersion into environmental waters for in situ measurements, providing a platform for dynamic analysis of microorganism–pollutant interactions and advancing water treatment and environmental monitoring technologies.

## Introduction

Yeast, particularly *Saccharomyces cerevisiae*, is characterized by a cell wall rich in polysaccharides such as β-glucans and mannans, as well as proteins [1–3]. Because these cell wall components contain a variety of functional groups, they can interact with diverse chemical substances and exhibit adsorption properties on the cell surface. For example, studies on antibiotics and aromatic organic compounds have reported that adsorption onto the yeast cell surface follows either the Langmuir adsorption isotherm or the Freundlich adsorption isotherm [4–6]. Further, quantitative structure–activity relationship analysis suggests that hydrophobic interactions, dispersion forces, and electrostatic interactions play important roles in adsorption onto the yeast surface.

Adsorption of metal ions by yeast has also been reported, and functional groups present in the cell wall, such as carboxyl, hydroxy, amino, and phosphate groups, are known to form electrostatic interactions and coordination bonds with metal ions. Indeed, the binding of divalent metal ions such as Pb^2+^, Zn^2+^, and Ni^2+^ to the yeast cell surface has been documented [7–9]. These adsorption behaviors are strongly influenced by solution conditions, particularly pH; in the pH range of 4–6, relatively high adsorption capacities are observed due to changes in the dissociation states of cell wall functional groups and the speciation of metal ions. Such diverse adsorption properties suggest a wide range of potential applications, including their use as drug carriers [10–11] and as adsorbent materials for pollutants as biosorption materials [12–13]. In environmental applications, the use of yeast biomass has been investigated for the removal of heavy metals from electroplating and mining wastewater [12,14–15], as well as organic pollutants such as dyes in textile effluents [16–17], and the development of yeast-based water purification technologies has been actively pursued.

Despite these advances, conventional methods for adsorption analysis have primarily relied on batch adsorption experiments [5], in which changes in solute concentration are evaluated using absorbance measurements or chromatographic analyses, and adsorption capacities are calculated under equilibrium conditions. Consequently, these approaches provide limited information on the time-resolved dynamics of adsorption and do not allow for direct observation of interfacial molecular interactions at the yeast surface. As a result, the dynamic adsorption behavior of drug molecules and pollutants on yeast has not been sufficiently elucidated. This limitation hampers a deeper understanding of adsorption kinetics and restricts the rational design of yeast-based materials for applications such as drug delivery and environmental remediation. To address these challenges, analytical platforms capable of directly monitoring adsorption processes at the bio-interface in real time are required. However, such approaches remain limited, particularly for systems involving complex biological surfaces such as yeast cells.

In this study, we developed an optical fiber-based surface plasmon resonance (SPR) sensor incorporating a yeast-derived bio-interface, enabling real-time and in situ monitoring of adsorption processes. In the fabrication of the SPR sensor, electroless Ni plating was performed while light from an LED was introduced into the optical fiber. The transmitted light intensity was measured using a spectrometer connected to the opposite end; by converting the obtained intensity into absorbance, we demonstrated that thin-film formation could be achieved while monitoring changes in film thickness in real time. The usefulness of this film thickness monitoring approach has been demonstrated in optical fiber SPR sensors incorporating Ag nanofilms [18]. This fabrication strategy improved the reproducibility and success rate of sensor preparation compared with conventional reflectance-based methods using bifurcated fibers [19]. After forming a gold nanofilm via Au displacement plating, yeast cells were successfully and uniformly immobilized on the gold surface by spraying a yeast suspension using an airbrush.

By integrating a yeast-based interface with an optical fiber SPR platform, the proposed system enables real-time monitoring of adsorption processes at biologically relevant interfaces, providing a useful tool for investigating molecular interactions at biological interfaces. Furthermore, the proposed platform has the potential to enable comprehensive evaluation of interactions between complex environmental samples and microbial cell surfaces. This approach provides a foundation for understanding such biological interfacial interactions.

## Results and Discussion

### Monitoring of film thickness during electroless Ni plating by absorbance measurement

**Figure 1:**
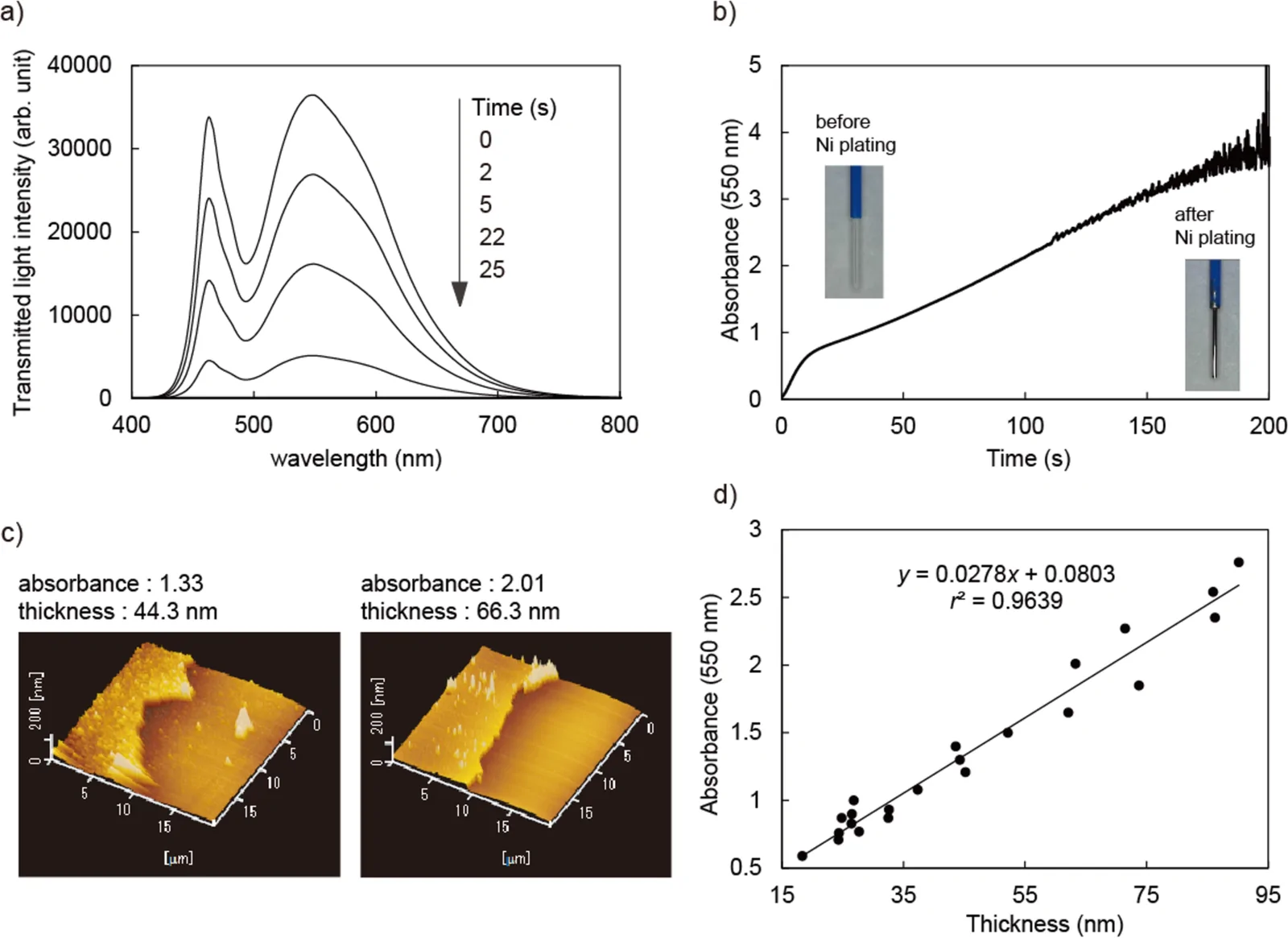
Formation of a gold thin film on an optical fiber core via electroless Ni plating. (a) Dependence of the transmitted light intensity through the fiber on the electroless plating reaction time. (b) Change in absorbance as a function of Ni deposition time. (c) Observation of Ni thin film thickness by AFM. (d) Correlation between absorbance and Ni film thickness.

**Figure 2:**
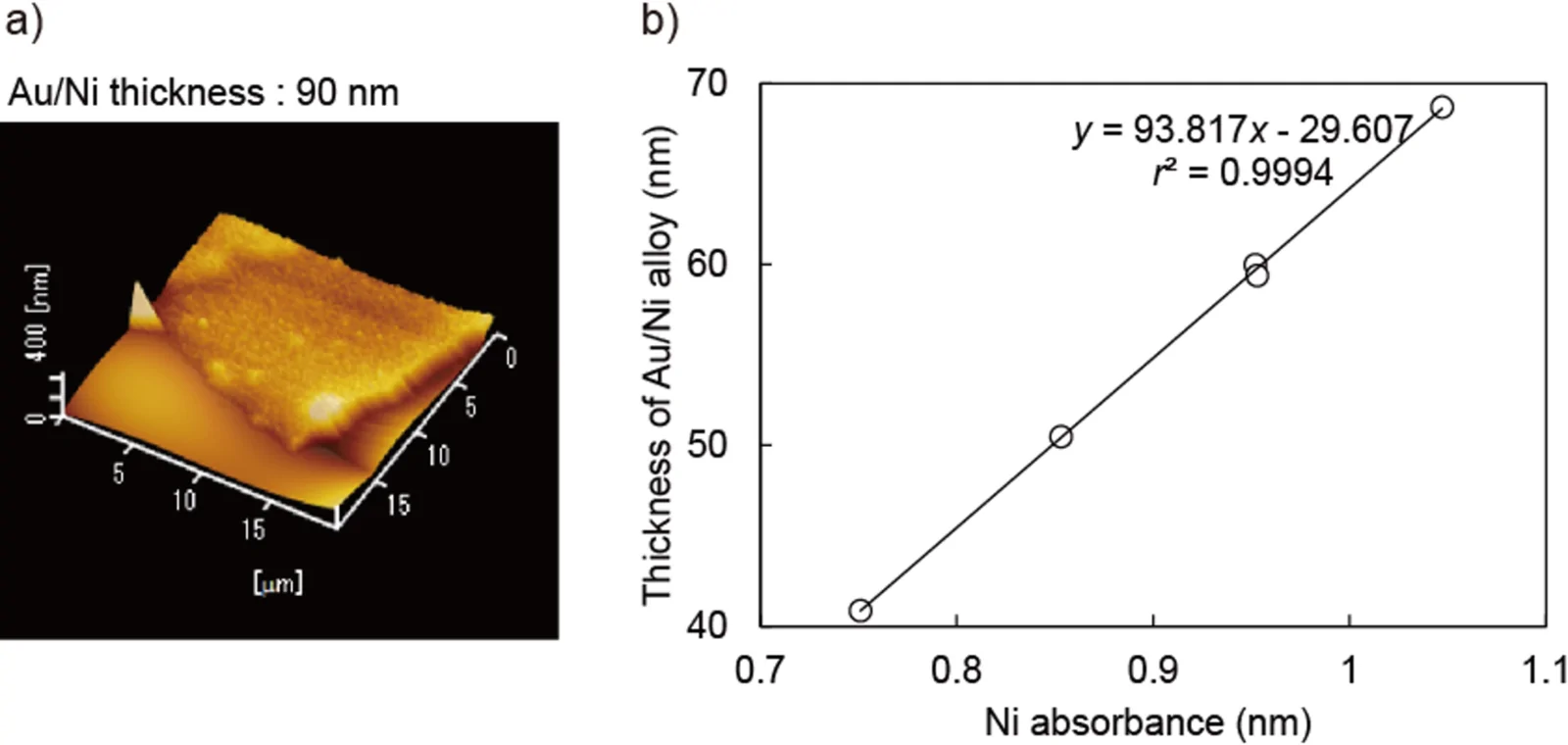
Formation of a gold thin film via electroless plating. (a) Thickness of the gold thin film observed by AFM. (b) Thickness of gold and Ni alloy thin films obtained by electroless gold plating, plotted as a function of absorbance during the electroless Ni plating reaction.

We have previously reported that the progress of the electroless plating reaction at the tip of an optical fiber can be monitored by reflectance measurements [19]. However, the reflected light intensity exhibited poor reproducibility, with signal fluctuations observed between measurements (Supporting Information File 1, Figure S1). This variability is likely due to differences in the cutting angle and surface roughness of the fiber tip, which affect the efficiency of reflected light collection. In this study, we investigated the use of absorbance, calculated from transmitted light intensity, to monitor the progress of the electroless Ni plating reaction. As the reaction proceeded, the reflected light intensity decreased (Figure 1a). The time course of absorbance showed a gradual increase with reaction time, corresponding to an increase in the thickness of the Ni thin film formed at the fiber tip (Figure 1b). These results indicate that the progress of the electroless Ni plating reaction can be monitored in real time by absorbance measurements. To evaluate the relationship between absorbance and the thickness of the deposited Ni thin film, a portion of the optical fiber surface was etched with dilute nitric acid to create a step, and the step height was measured by atomic force microscopy (AFM) (Figure 1c). The results showed that higher absorbance values corresponded to larger step heights, indicating an increase in Ni film thickness. To further examine this relationship, absorbance was plotted against Ni film thickness, revealing a strong linear correlation, as shown in Figure 1d (*y* = 0.0278*x* + 0.0803, *r*^2^ = 0.9639). This finding demonstrates that absorbance measurements can be used to monitor the thickness of Ni thin films formed during electroless plating. To form a gold thin film suitable for SPR measurements, Ni/Au displacement plating was subsequently performed. In this process, Ni was dissolved while Au was simultaneously deposited and grew on the fiber surface. Because Au has a larger atomic radius than Ni, the thickness of the resulting Au/Ni film formed by displacement plating may differ from that of the initial Ni thin film. Therefore, the relationship between the Ni film thickness before displacement and the Au/Ni film thickness after displacement was examined. As shown in Figure 2, a strong linear correlation was obtained between the two *(y* = 93.817*x* − 29.607, *r*^2^ = 0.9994). In general, a gold thin film with a thickness of approximately 50 nm is considered optimal for SPR measurements. Based on this relationship, it was found that allowing the electroless Ni plating reaction to proceed until the absorbance reaches approximately 0.85 enabled the formation of a gold thin film with a thickness suitable for SPR sensing.

### Performance evaluation of the SPR platform fabricated by absorbance-based thickness monitoring

**Figure 3:**
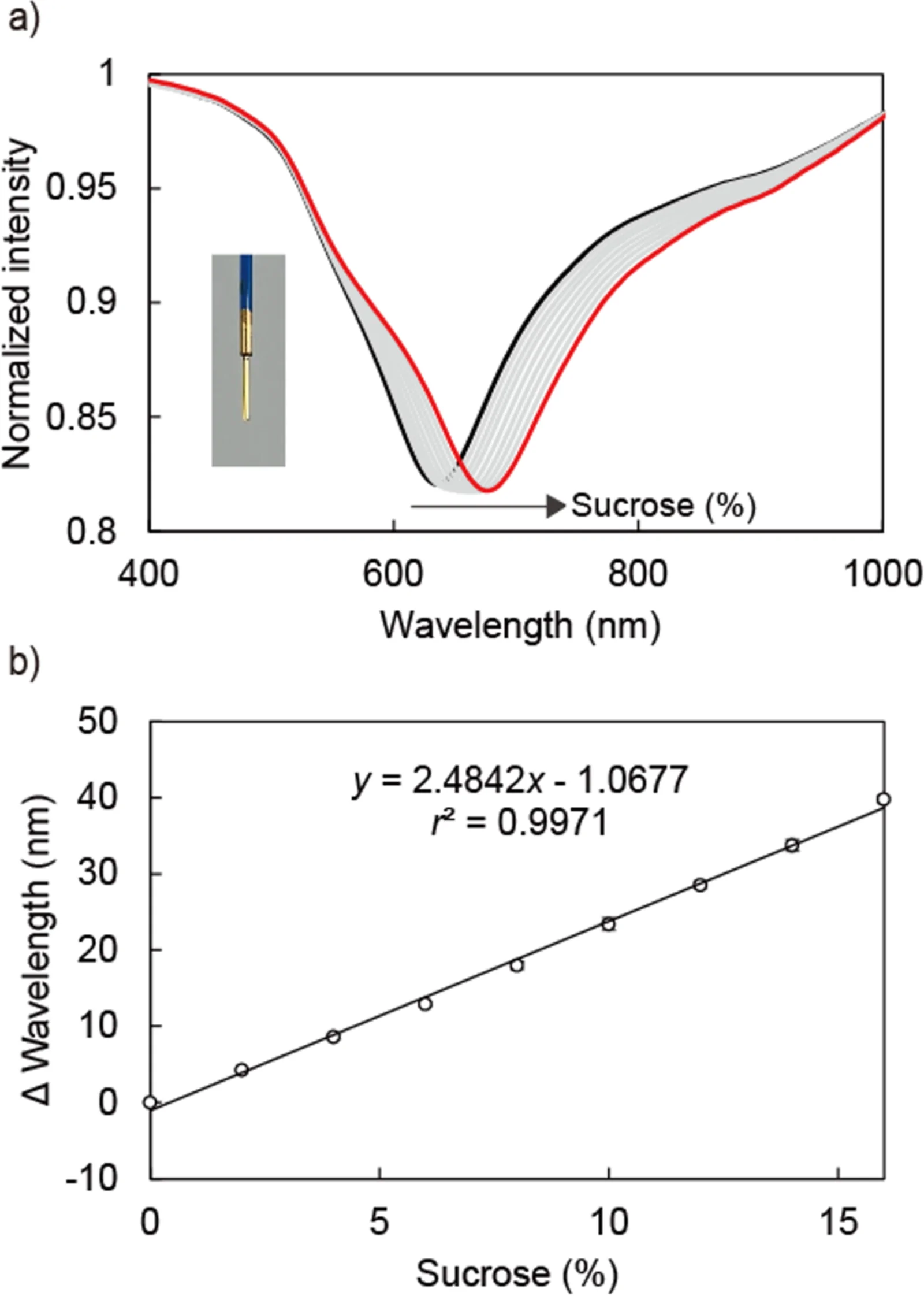
Performance evaluation of the optical fiber surface SPR sensor. (a) Dependence of the SPR spectrum on sucrose concentration; the inset shows the optical fiber SPR sensor. (b) Shift in SPR resonance wavelength as a function of sucrose concentration, calculated with the resonance wavelength obtained in water defined as 0 nm. Error bars represent the standard deviation (*n* = 3).

To evaluate the performance of the optical fiber SPR sensor fabricated using absorbance-based thickness monitoring, SPR spectra were measured using aqueous sucrose solutions. As the sucrose concentration increased, the resonance wavelength of the SPR spectrum exhibited a shift toward longer wavelengths (red shift) (Figure 3a). This behavior is attributed to an increase in the refractive index of the solution with increasing sucrose concentration, which alters the SPR resonance condition. A linear relationship was observed between the resonance wavelength and sucrose concentration, with *y* = 2.484*x* + 1.068, *r*^2^ = 0.997 (Figure 3b). The fabricated SPR sensor showed a good linear response over a refractive index range of 1.333–1.357, with a sensitivity of 1710 nm/RIU. The performance of the fabricated SPR sensor was compared with that of previously reported optical fiber SPR sensors fabricated by other methods. Optical fiber SPR sensors fabricated by sputtering have been reported to exhibit a linear range of 1.3342–1.3762 and a sensitivity of 1959 nm/RIU [20]. In contrast, sensors fabricated by vacuum evaporation exhibit a linear range of 1.333–1.3469 and a sensitivity of 1557 nm/RIU [21]. Compared with these reports, the optical fiber SPR sensor fabricated in this study using absorbance-based thickness monitoring demonstrates a comparable linear range and sensitivity to those of sensors prepared by conventional thin-film deposition methods, indicating that the present approach provides performance on par with established fabrication techniques.

### Yeast immobilization on a gold nanofilm

**Figure 4:**
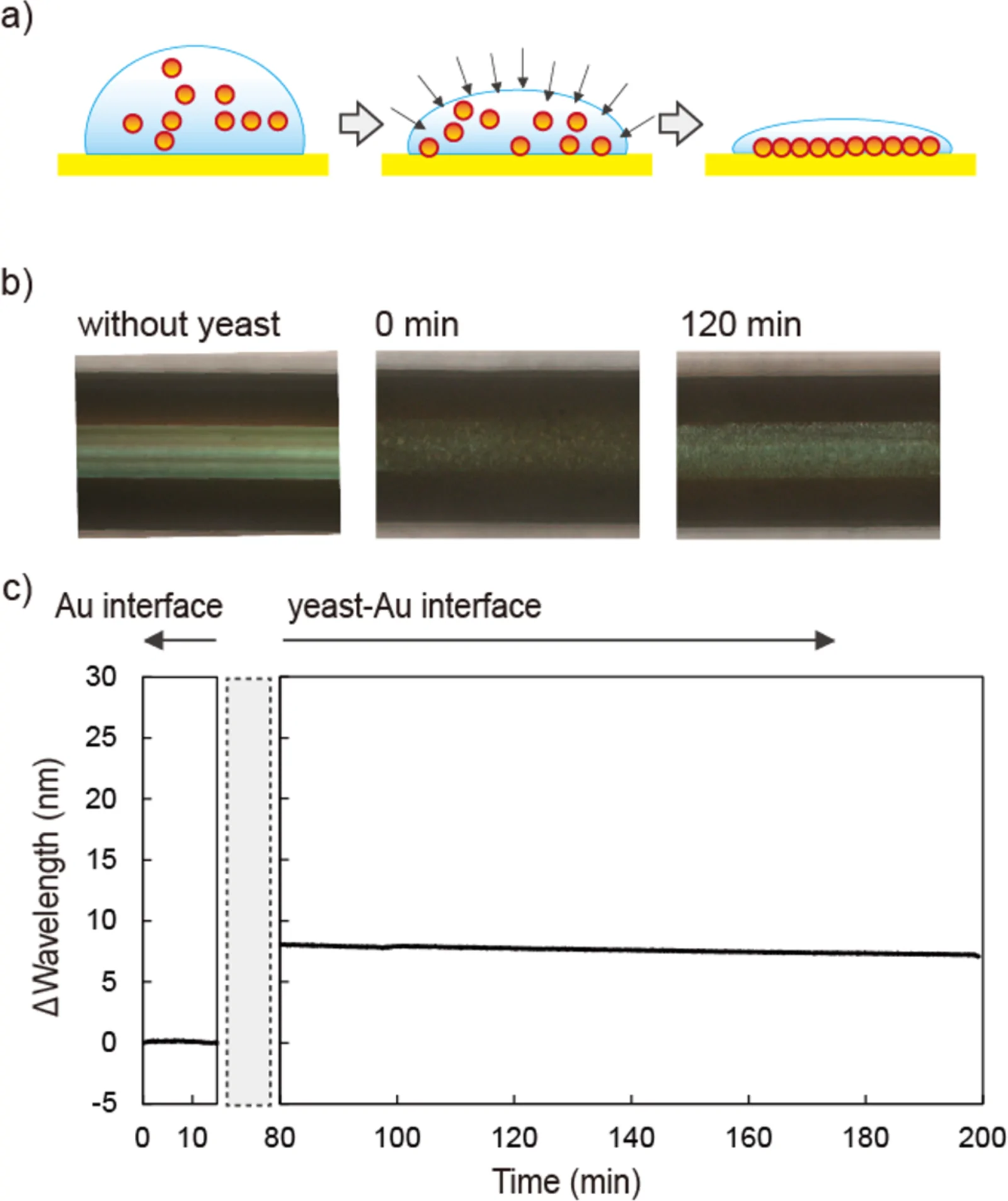
Immobilization of yeast on the gold surface of an optical fiber SPR sensor pre-adsorbed with lysozyme. (a) Schematic illustration of the immobilization method. Arrows indicate the decrease in water content due to evaporation, and circles represent yeast cells. (b) Process of yeast immobilization onto the lysozyme-functionalized fiber surface. (c) Change in SPR resonance wavelength before and after yeast immobilization. The gray square indicates the spraying step of fine droplets of yeast suspension using an airbrush; the resonance wavelength was not recorded during this process.

The method for immobilizing yeast cells on the optical fiber SPR sensor interface was investigated. A strategy was adopted in which proteins were physically adsorbed onto the gold nanointerface, and yeast cells were subsequently immobilized via chemical linkage through amino groups. Bovine serum albumin (BSA) and lysozyme were considered as candidate proteins. BSA is known to bind various pharmaceutical compounds through hydrophobic pockets corresponding to two major drug-binding sites (Sudlow sites I and II) [22]. Therefore, interactions between pharmaceuticals and BSA could interfere with the evaluation of adsorption behavior onto yeast. In contrast, lysozyme has been reported to bind several compounds more weakly than BSA [23–25]. Based on these considerations, lysozyme was selected for immobilization on the gold nanointerface in this study. After physically adsorbing lysozyme onto the gold nanointerface, the surface was activated by introducing glutaraldehyde, followed by immersion in a yeast suspension. However, because yeast cells gradually sediment over time, sufficient contact between the cells and the sensor interface could not be maintained, resulting in inefficient immobilization onto the gold nanointerface. To address this issue, a spray-based immobilization method using an airbrush was investigated. In this approach, fine droplets gradually shrink as the solvent evaporates at room temperature, leading to concentration of yeast cells within the droplets (Figure 4a). As a result, yeast cells become concentrated on the gold nanointerface and are immobilized via the aldehyde groups of glutaraldehyde immobilized on the lysozyme-coated surface (Figure 4b). Upon yeast immobilization, the SPR signal increased by approximately 8 nm (Figure 4c). Furthermore, the stability of the immobilized yeast layer was evaluated by immersing the optical fiber SPR sensor with the yeast bio-interface vertically in water. The results showed that the yeast cells remained on the interface over a measurement period of 2 h, with no significant decrease in the SPR signal (Figure 4c). These findings demonstrate that the proposed spray immobilization method enables stable immobilization of yeast cells on the optical fiber SPR sensor interface.

### Interaction between the compound and the yeast cells surface

Knowledge regarding pharmaceutical adsorption by yeast remains limited, and a systematic understanding of the adsorption mechanisms, selectivity, and their relationships with molecular structure has not yet been fully established. Consequently, design guidelines for yeast-based materials as adsorbents for contaminant removal remain to be further developed. Yeast cell walls are composed of multiple biopolymers, including chitin, β-glucans, and mannoproteins, which together form a structurally and chemically complex three-dimensional architecture. Therefore, interactions between drug molecules and the cell wall cannot be attributed to a single component. In this context, this study does not aim to deconvolute the contributions of individual components but instead focuses on establishing a yeast-based interfacial sensing platform, for which chitin, a structurally stable component, was employed.

**Figure 5:**
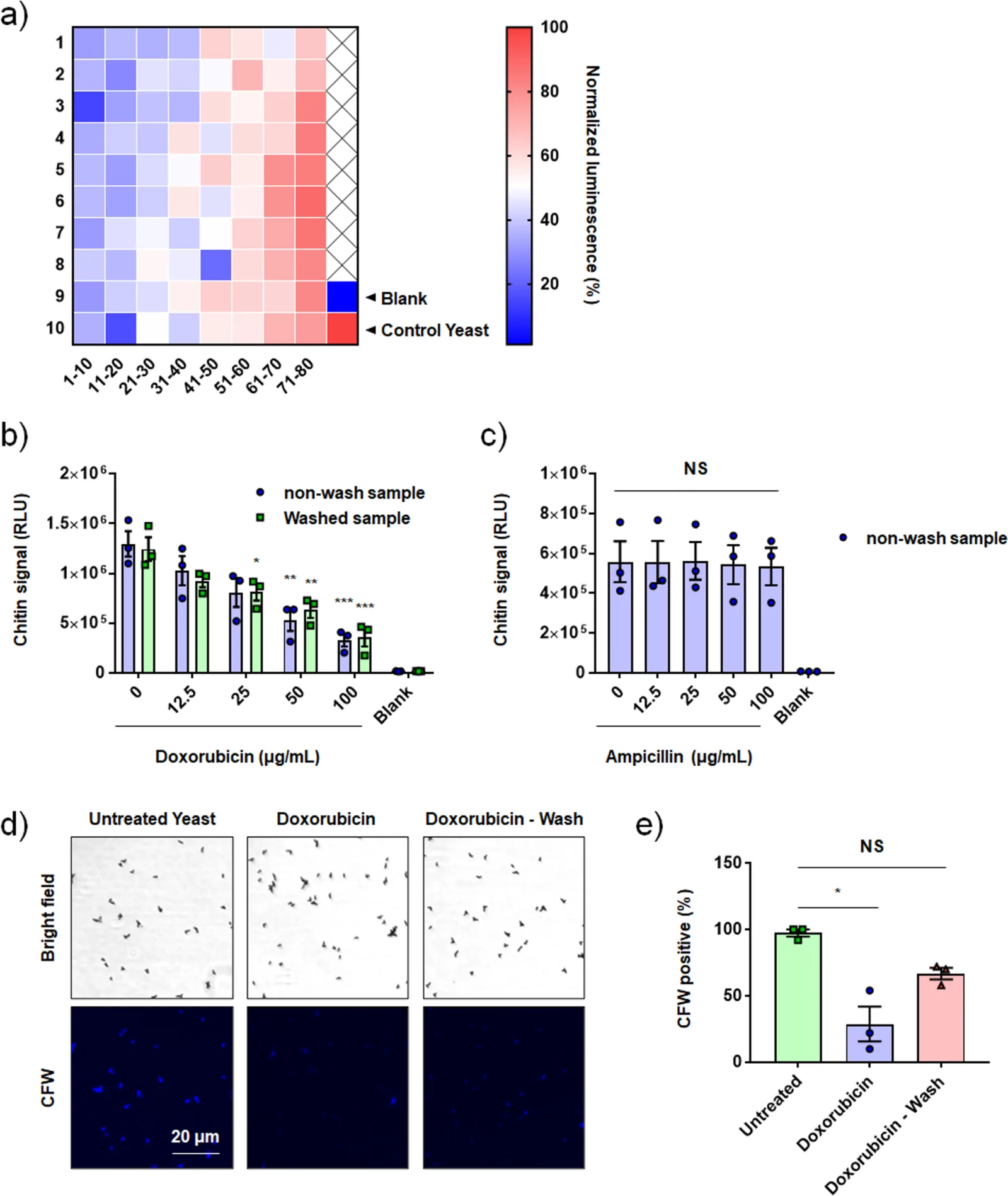
Screening of compounds that interact with cell wall chitin. (a) Cell surface chitin signals from compounds treated HKSc. The luciferase activity of control yeasts normalized to the luminescence of cell-free blank wells was set to 100%, with increased samples shown in red and decreased samples shown in blue. (b, c) Chitin signals from HKSc treated with (b) doxorubicin or (c) ampicillin (0–100 μg/mL, final). Blank indicates wells without yeast. Histograms show mean relative surface chitin ± SEM (*n* = 3). *p < 0.05, **p < 0.01, ***p < 0.001: significant difference vs control yeast (One-way ANOVA with Bonferroni’s multiple test). (d) Microscopy images of total chitin stained with CFW in doxorubicin-treated yeast cells. (e) CFW-positive rate in doxorubicin-treated cells. Histograms show mean CFW-positive rate ± SEM (*n* = 3). *p < 0.05: significant difference vs untreated yeast (One-way ANOVA with Dunn’s multiple test).

To screen for compounds that directly bind to the yeast cell wall, heat-killed yeast cells were prepared and mixed with an antibiotic-focused compound library (80 compounds). In this study, we focused on chitin, which is water-insoluble and stably present in the fungal cell wall, and performed non-wash chitin detection based on the split-luciferase complementation (SLC) assay using a chitin-specific binding protein. In this assay, inhibition rates were calculated based on changes in luciferase signals following compound treatment and visualized using a color scale (Figure 5a). Compounds shown in blue exhibit reduced luciferase signals, which may reflect decreased accessibility of cell surface chitin or masking of chitin-binding sites. In this context, these compounds may interact with cell wall chitin, thereby reducing its detectability in the assay. In contrast, compounds shown in red indicate increased signals, suggesting enhanced exposure or detectability of cell surface chitin.

In our compound library, compound no. 3, doxorubicin hydrochloride, reduced the surface chitin-derived signal by more than 20% (Figure 5a). In contrast, for example, compound no. 63, ampicillin trihydrate, which did not strongly suppress the chitin signal, was considered to have low affinity for the yeast cell wall and was used as a negative control in subsequent experiments. To verify that the results observed in the SLC assay were not due to inhibitory substances derived from yeast cells that could potentially affect luciferase activity, yeast cells were incubated with various concentrations of doxorubicin. The cells were then washed three times to remove unbound compounds and potential interfering substances, after which the assay was performed. As a result, even after thorough washing, the chitin signal was significantly and dose-dependently reduced with increasing concentrations of doxorubicin (Figure 5b). In contrast, no change in the chitin signal was observed even at increasing concentrations of ampicillin (Figure 5c). These results suggest that cell surface chitin may be masked by doxorubicin. To further validate this, live yeast cells were treated with doxorubicin, and total cell wall chitin was stained using a low-molecular-weight chitin-binding dye, Calcofluor white (CFW). Quantification of CFW-positive cells revealed a significant decrease following doxorubicin treatment, while washing the treated cells increased the number of positive cells (Figure 5d,e). This result supports an interaction between doxorubicin and cell wall chitin.

### Real-time monitoring of pharmaceutical adsorption on the yeast bio-interface

From the screening results, doxorubicin, which showed pronounced adsorption onto yeast, and ampicillin, which exhibited negligible adsorption, were selected as model compounds, and their adsorption behavior was evaluated by SPR measurements. Importantly, doxorubicin can enter the environment through hospital effluents and wastewater treatment processes. It is known that conventional wastewater treatment does not completely remove such compounds [26]. Doxorubicin, along with other anticancer agents, has been increasingly discussed in relation to emerging environmental contaminants due to concerns regarding persistence and potential toxicity to aquatic organisms [27].

**Figure 6:**
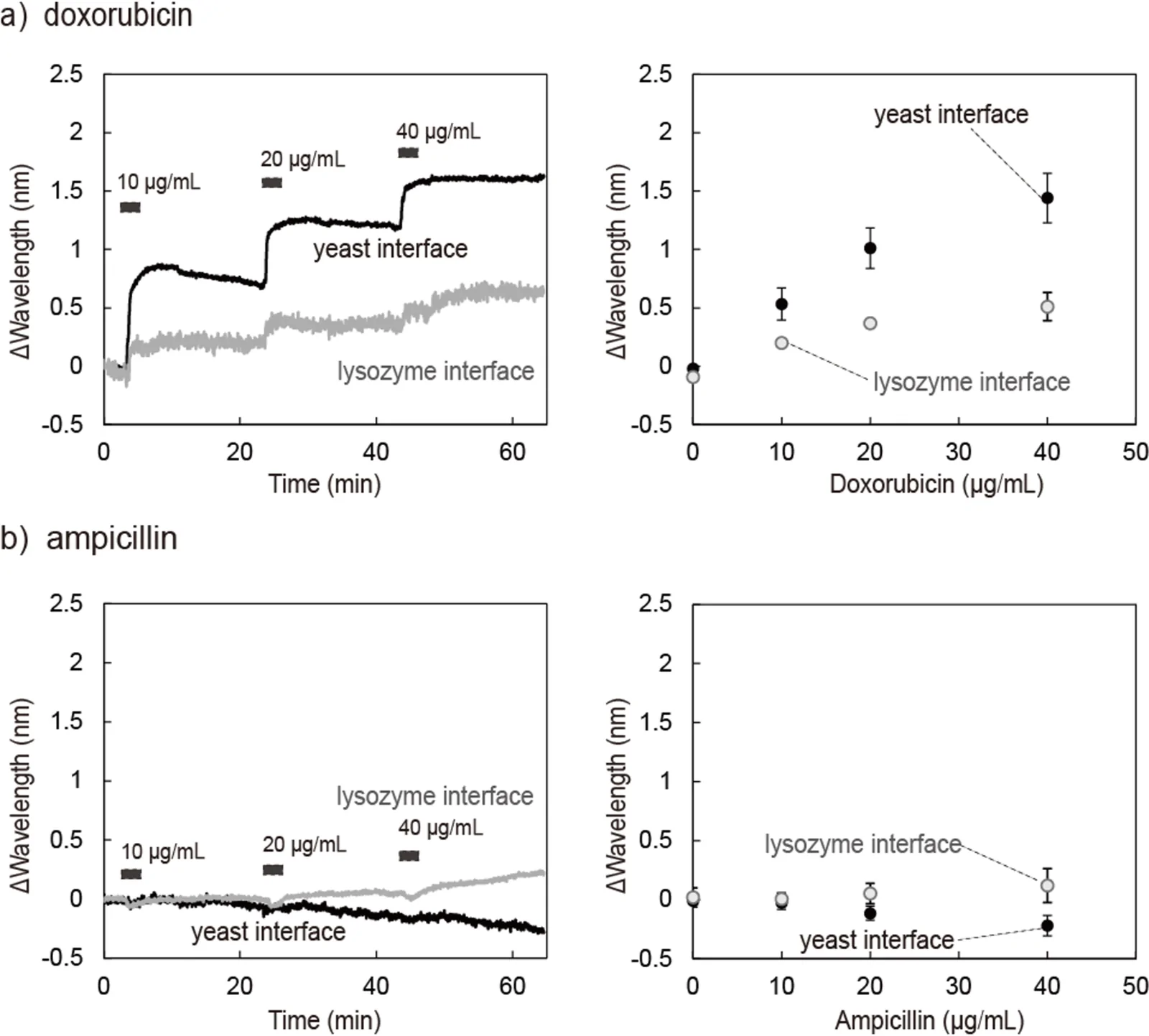
Responses of the optical fiber SPR sensor with a yeast-immobilized interface to (a) doxorubicin and (b) ampicillin. The left panels show sensorgrams representing changes in SPR resonance wavelength upon analyte addition; the black line corresponds to the yeast-immobilized optical fiber SPR sensor, while the gray line represents the lysozyme-functionalized optical fiber SPR sensor. Black squares indicate the time points of yeast suspension addition, and the concentrations shown correspond to the final concentrations after addition. The right panels show the dependence of the resonance wavelength shift on pharmaceuticals concentration. Error bars represent the standard deviation (*n* = 3).

The optical fiber SPR sensor with the yeast bio-interface was immersed in ultrapure water, followed by stepwise addition of a doxorubicin solution. Upon addition of doxorubicin, the SPR signal increased immediately (Figure 6a). Furthermore, with increasing doxorubicin concentration, the SPR signal measured 20 min after addition, corresponding to the equilibrium value, also increased (Figure 6b). These results indicate that doxorubicin adsorbs onto the yeast surface in a concentration-dependent manner. In contrast, no significant increase in the SPR signal was observed for the lysozyme interface without immobilized yeast. Doxorubicin is composed of an anthracycline-based aromatic polycyclic structure and a sugar moiety containing an amino group. These structural features may enable multiple interactions with the yeast cell wall. For example, the aromatic polycyclic structure might facilitate hydrophobic interactions with hydrophobic regions of the cell wall, while the amino group may become protonated under near-neutral pH conditions, potentially enabling electrostatic interactions with negatively charged sites on the yeast surface. In addition, the aromatic rings may form π–π interactions with aromatic residues in cell wall proteins. The cooperative contribution of these interactions may be responsible for the relatively strong adsorption of doxorubicin onto the yeast surface. However, the proposed interaction mechanism remains speculative and is not directly supported by experimental evidence in this study. In contrast, when ampicillin was added, no significant increase in the SPR signal, as observed for doxorubicin, was detected (Figure 6c,d). This result indicates that ampicillin exhibits little adsorption onto the yeast surface, consistent with the screening results obtained using the chemiluminescence probe. Ampicillin is a relatively small molecule with a β-lactam structure and contains both carboxyl and amino groups; however, it is overall highly hydrophilic. Moreover, because ampicillin lacks an extended hydrophobic surface such as an aromatic polycyclic structure, it is expected to be less likely to form hydrophobic interactions with the cell wall. In addition, under near-neutral pH conditions, ampicillin predominantly exists as a zwitterion or partially negatively charged species, which may weaken electrostatic interactions with negatively charged sites on the yeast surface. These findings demonstrate that the yeast bio-interface-type optical fiber SPR sensor enables real-time evaluation of the adsorption behavior of pharmaceuticals onto yeast.

## Conclusion

In this study, we established a method for monitoring film thickness during the electroless Ni plating process in the fabrication of an optical fiber SPR sensor by measuring the absorbance of transmitted light. A strong linear relationship was obtained between absorbance and the thickness of the Ni thin film, demonstrating that absorbance measurements enable reproducible formation of gold thin films suitable for SPR sensing. The optical fiber SPR sensor fabricated using this method exhibited sensitivity and a linear response range comparable to those of sensors prepared by conventional sputtering and vacuum evaporation methods when evaluated using sucrose solutions. These results indicate that a high-performance SPR sensor can be constructed through a simple plating process. Further, a spray-based immobilization method was proposed for yeast fixation onto the gold nanointerface, enabling the construction of a yeast bio-interface-type optical fiber SPR sensor. Using this sensor, the adsorption behavior of pharmaceuticals onto yeast was successfully monitored in real time. In particular, a concentration-dependent increase in the SPR signal was observed for doxorubicin, whereas no significant signal change was detected for ampicillin. These findings suggest that the interactions between yeast cell walls and pharmaceutical molecules depend on molecular structure and charge state.

In the future, the proposed method is expected to be applicable to in situ measurements in environmental waters, such as rivers and lakes. In natural aquatic environments, a wide variety of chemical constituents coexist and interact competitively with the yeast cell wall. Because faithfully reproducing such complex interfacial interactions under laboratory conditions is challenging, the proposed method has the potential to enable the direct evaluation of adsorption behavior in real environmental waters. Furthermore, the selectivity of the platform can be tailored by changing the immobilized microorganism. Different microorganisms possess distinct cell wall architectures and surface properties, resulting in different interaction profiles with chemical substances, potentially enabling the evaluation of a broader range of molecule–cell wall interactions. The proposed platform is expected to serve as a biological screening tool for evaluating the overall interactions between complex environmental samples and microorganism surfaces.

## Experimental

### Chemicals and materials

Unless otherwise specified, all reagents used in this study were of analytical grade. Dilute nitric acid, sulfuric acid, ethanol, glycine, acetone, silica gel, 0.1 M hydrochloric acid, sucrose, sodium dihydrogen phosphate, and disodium hydrogen phosphate were purchased from Kanto Chemical Co., Inc. (Tokyo, Japan). Hydrogen peroxide, Ni(II) sulfate hexahydrate, trisodium citrate dihydrate, 3-aminopropyltrimethoxysilane (APS), anhydrous toluene, palladium(II) chloride, sodium borohydride, 10% glutaraldehyde solution, ampicillin, Dulbecco’s phosphate-buffered saline (D-PBS) without calcium and magnesium, dimethyl sulfoxide (DMSO), and Tween 20 were purchased from FUJIFILM Wako Pure Chemical Corporation (Osaka, Japan). Bovine serum albumin (BSA), Calcofluor white (CFW), and sodium hypophosphite were obtained from Sigma-Aldrich (St. Louis, MO, USA). Sodium dodecyl sulfate (SDS), lysozyme from chicken egg, and doxorubicin hydrochloride were purchased from Tokyo Chemical Industry Co., Ltd. (Tokyo, Japan). PlusOne Tris was purchased from Amersham Biosciences. An antimicrobial compound library consisting of 80 compounds dissolved in DMSO at a stock concentration of 10 mM (Supporting Information File 1, Table S1) was purchased from Selleck Chemicals (Houston, TX, USA). The gold plating solution used for nickel-to-gold displacement plating was kindly provided by HIKIFUNE Co., Ltd. All water used in all experiments was purified using a PURELAB Flex 3 system (Veolia Jenets K.K., Tokyo, Japan).

### Real-time monitoring system for Ni thin-film formation

A system for real-time monitoring of the thickness of Ni nanofilms formed by electroless plating is shown in Figure 1a. The system consists of a power LED (OSW4XNE3C1E, OptoSupply Ltd., Hong Kong, China), a custom-built power supply for the LED, a bare fiber adapter (BFA-KIT, Ocean Insight, FL, USA), an optical fiber cable (M28L01, Thorlabs Inc., NJ, USA), and a spectrometer (USB4000, USB4E03214, Ocean Insight, FL, USA). In addition, a microheater, a power supply for heating, a relay, and a temperature controller were used to heat the Ni plating solution. Light emitted from the power LED was directed onto the end face of the fiber core, and the transmitted light emitted from the opposite end was guided through the optical fiber cable and detected by the spectrometer. The transmitted light intensity was measured at intervals of 0.1 s, and the monitoring wavelength was set at 550 nm. The transmitted light intensity was converted into absorbance using dedicated software, Optical Fiber SPR Monitor, Version 1.0.3.0 (Kyusyu Keisokki Co., Ltd., Fukuoka, Japan). The absorbance (−log *I_t_*/*I*_0_) was calculated from the ratio of the transmitted light intensity at time *t* (*I_t_*) to that at the start of the Ni plating reaction (*I*_0_).

### Amination of optical fiber surface

An optical fiber with a core diameter of 400 µm (FT400EMT, Thorlabs Inc., NJ, USA) was cut to a length of approximately 130 mm, and both end faces were polished to a smooth finish using a fiber polishing machine (Radian TrigLTTM, KRELLTECH, NJ, USA). A section of the jacket approximately 5 mm in length was removed from one end using a fiber buffer stripper (T21S31, Thorlabs Inc., NJ, USA), and the cladding was subsequently removed with acetone-soaked cotton to expose the fiber core. The exposed fiber core was immersed in a piranha solution prepared by mixing hydrogen peroxide and sulfuric acid at a volume ratio of 1:3, followed by thorough rinsing with ultrapure water. The cleaned fiber was then dried for more than 1 h in a constant-temperature oven set at 120 °C (OFW-300V-R, AS ONE, Osaka, Japan). To introduce amino groups onto the fiber core surface, the exposed core was immersed in a 1.0% (v/v) solution of 3-aminopropyltrimethoxysilane (APS) prepared in toluene, and incubated at 40 °C with stirring at 100 rpm. After 1 h, the fiber was removed from the APS solution and dried at room temperature.

### Monitoring of absorbance changes during electroless Ni plating

**Figure 7:**
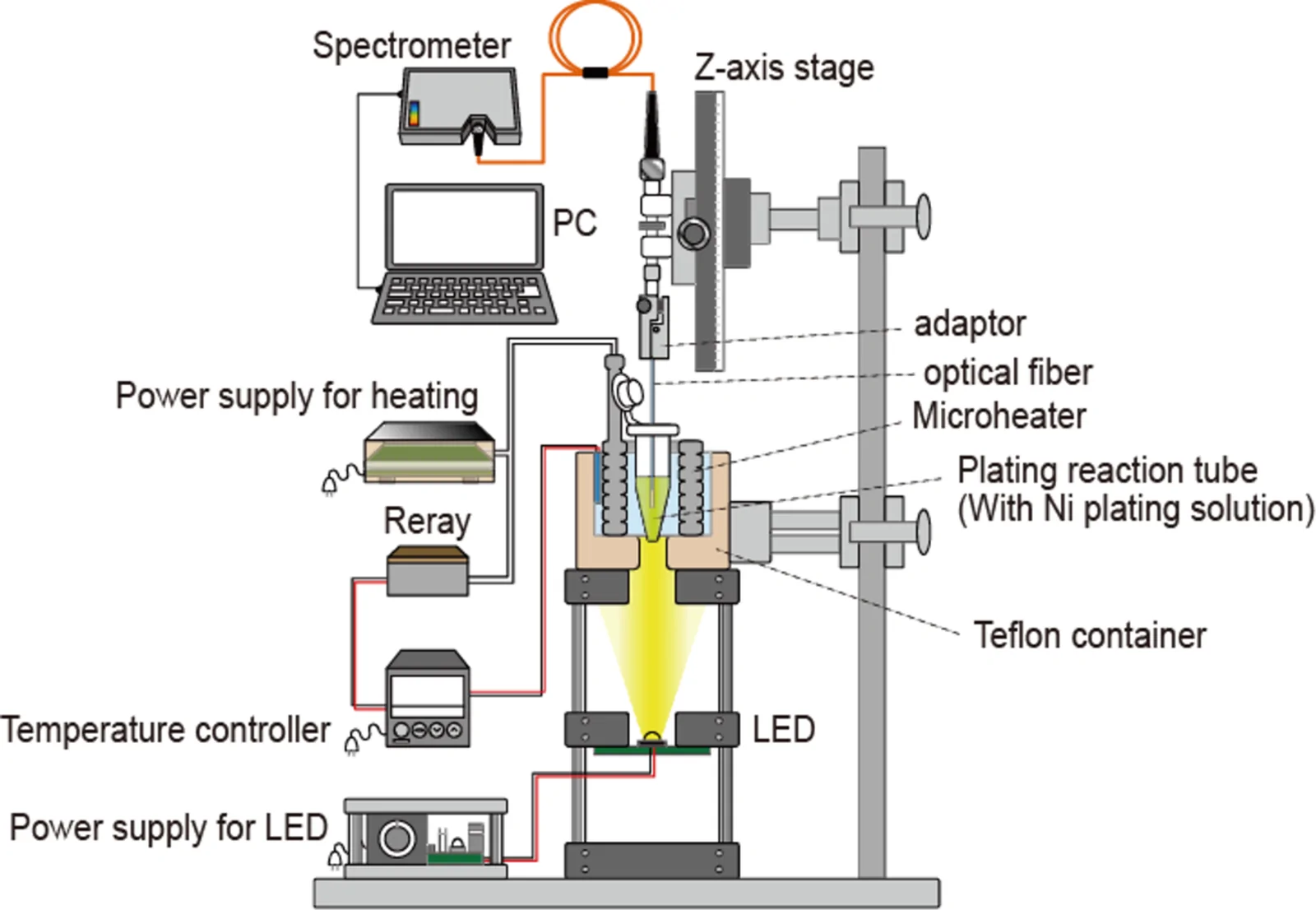
Schematic illustrations of the experimental setups. Monitoring system for the electroless Ni plating reaction.

A Ni plating solution was prepared with ultrapure water containing 110 mM Ni(II) sulfate hexahydrate, 93 mM sodium hypophosphite, 270 mM glycine, 93 mM trisodium citrate dihydrate, and 0.5% (v/v) SDS. One milliliter of this solution was placed in a glass vial and heated to 85 °C using a coil connected to a heating unit (Figure 7). The exposed fiber core was first immersed in a 0.5% (w/v) Pd solution prepared in 0.1 M hydrochloric acid for 3 min at room temperature to adsorb Pd^2+^ ions, followed by immersion in a 0.1% (w/v) sodium borohydride solution prepared in ethanol for 3 min. Subsequently, the optical fiber was immersed in the Ni plating solution maintained at 85 °C to initiate plating. The absorbance was calculated from the transmitted light intensity immediately after immersion (*I*_0_) and at a given time *t* (*I_t_*), and the change in absorbance was monitored in real time. Electroless Ni plating was continued until the absorbance reached a value of 0.85.

### Monitoring of the thickness of gold nanofilms formed by electroless Ni/Au plating

A volume of 1.0 mL of Au plating solution was transferred into a glass vial and heated to 84 °C. An optical fiber coated with a Ni film was immersed in the heated gold plating solution, and the Ni thin film was replaced with a gold thin film via a galvanic displacement reaction based on the difference in ionization tendency. The thickness of the Au layer depends on that of the underlying Ni thin film; therefore, electroless Ni/Au plating was conducted for 2 min, with an additional 25 s of reaction applied if necessary.

### Thickness measurement of metal thin films formed by electroless plating

The thickness of the Ni film formed on the optical fiber core surface by electroless plating was evaluated by step-height measurement using an atomic force microscope (AFM5100N, Hitachi High-Technologies Co., Ltd., Tokyo, Japan). For the measurement, a portion of the Ni thin film on the optical fiber surface was chemically dissolved and removed using a dilute nitric acid solution to expose the underlying glass substrate. Subsequently, an area including both the Ni film surface and the exposed glass surface was scanned by AFM in tapping mode, and the height difference between the Ni surface and the glass surface was determined as the step height. The height profile was obtained by line profile analysis of the acquired AFM images, and the average height difference between the Ni film surface and the glass substrate surface was defined as the film thickness. Measurements were performed at multiple locations, and the average of the obtained values was reported as the thickness of the Ni film.

### Evaluation of the fabricated sensor using sucrose measurements

The performance of the fabricated sensor was evaluated using the optical fiber SPR measurement system [18]. The system consists of a halogen lamp (KBEX-151B, Nissei Electric Co., Shizuoka, Japan), a bifurcated optical fiber (Thorlabs), and a spectrometer, to which the fabricated optical fiber SPR sensor was connected for measurements. Light emitted from the halogen lamp was guided to the sensor through the bifurcated fiber, and the light reflected from the sensor was detected by the spectrometer via the same fiber. The reflected light intensity obtained from the sensor immersed in the sample solution was normalized by the reflected light intensity measured in air to obtain the SPR spectrum. The performance of the fabricated sensor was evaluated using aqueous sucrose solutions with concentrations up to 16% (w/v). The minimum of the obtained SPR spectrum was defined as the SPR wavelength, and the shift in SPR wavelength (ΔSPR wavelength) with changing sucrose concentration was defined as the SPR signal. Furthermore, the sensor response was evaluated by plotting the SPR signal as a function of sucrose concentration.

### Yeast and culture

Brewer’s yeast, *Saccharomyces cerevisiae* strain NBRC 10217, was purchased from NITE biological resource center (NBRC, Chiba, Japan). Yeast cells maintained on potato dextrose (PD) agar were suspended in D-PBS and inoculated into a 50 mL conical tube containing 15 mL of PD medium and incubated for 24 h at 30 °C with shaking (200 rpm, BR-23FP Bioshaker, TAITEC Co., Saitama, Japan). The cells were then washed with water, and an aliquot was heat-treated at 90 °C for 10 min to obtain heat-killed yeast cells (HKSc).

### Yeast cell wall analysis

HKSc suspended in water (1 × 10^8^ yeast cells/mL) was mixed with antimicrobial compound library (100 μM, final), newly purchased compounds in water, or control (1 v/v % DMSO or water), incubated for 30 min at 30 °C and heat-treated at 90 °C for 10 min. To assess dose-dependent responses, samples were also prepared in which cells were washed with water after drug treatment to remove free compound. Cell surface-exposed chitin amount was evaluated using a split-luciferase complementation (SLC) assay, as described previously [28]. In brief, yeast suspension (1 × 10^8^ yeast cells/mL) was mixed in equal volume of chitin-specific protein probes (SmBiT- or LgBiT-fused hChit1-E140Q, 100 nM each) in assay buffer (PBS containing 1% BSA and 0.05% Tween 20). Reaction mixture was incubated in a 384-well white microplates (Greiner Bio-one, Frickenhausen, Germany) with shaking (400 rpm). After 30 min, luciferase substrate (Furimazine, Promega, WI, USA) was added to each well and reconstituted NanoLuc activity based on surface chitin amount was measured using a microplate reader (GloMax Discover Microplate Reader, Promega).

### Fluorescence microscopy images

Live yeast cells suspension in water (2 × 10^6^ yeast cells/mL) was mixed with doxorubicin hydrochloride (dissolved in water, 10 μg/mL, final), incubated for 1 h at 30 °C, followed by either no washing or three washes with pure water. For the total chitin staining, yeast cells were treated with 10 μg/mL of CFW solution and observed using a microscope equipped with DAPI filter (EVOS M5000 Imaging System, Thermo Fisher Scientific, MA, USA). Fifty yeast cells were randomly selected from bright-field images, and the proportion of CFW-positive cells was determined in the corresponding fluorescence images of the same field.

### Statistical analysis

All statistical analyses were performed using GraphPad Prism 7.0 (GraphPad Software, CA, USA). Based on the results of the normality test, significant differences were analyzed by one-way ANOVA followed by multiple comparisons using Dunn’s test or Bonferroni’s test.

### Fabrication of a yeast bio-interface-type optical fiber SPR sensor

To form a protein layer on the surface of the optical fiber SPR sensor, the fiber was first immersed in 10 mM phosphate buffer (PB, pH 7.4), and stabilization of the SPR signal was confirmed. Subsequently, a lysozyme solution at 200 µg/mL prepared in 10 mM PB (pH 7.4) was added to the PBS to achieve a final concentration of 10 µg/mL. The sensor was incubated in the solution until the SPR wavelength shift before and after lysozyme addition reached approximately 3 nm. Afterward, the sensor was immersed in a 1% glutaraldehyde (GA) solution (50 mM PB, pH 7.4) for 1 h to introduce aldehyde groups onto the surface. A yeast suspension with a cell concentration of 1.5 × 10^5^ yeast cells/mL was sprayed onto the GA-treated optical fiber SPR sensor surface using an airbrush, handpiece, connected to an oil-free air compressor (PROFIX NITRO-COMP V1, GSI Creos, Japan), generating fine droplets. After spraying, the sensor was left to stand for 1 h to allow immobilization of the yeast cells, followed by washing with water to remove non-immobilized cells. The sensor then underwent a blocking treatment by immersion in 10 mM Tris buffer (pH 7.4) for 20 min. Finally, the sensor was left to stand in 85 µL of water for 10 min to stabilize the sensor interface.

### Real-time measurement of pharmaceutical adsorption onto yeast

The yeast bio-interface-type optical fiber SPR sensor was immersed in 85 µL of water, and after stabilization of the SPR signal, 5 µL aliquots of doxorubicin or ampicillin solutions prepared in water were sequentially added to achieve final concentrations of 10, 20, and 40 µg/mL. The SPR signal after the addition of the pharmaceuticals was recorded in real time.

## Supporting Information

File 1 Additional figure and table.

## Data Availability

Data generated and analyzed during this study is available from the corresponding author upon reasonable request.
