# Supplementary material for: Design of a yeast-based bio-interface on an optical fiber SPR platform toward drug adsorption monitoring
Source: Beilstein J Nanotechnol. 2026 Sep 22;17:1290–301. doi: 10.3762/bjnano.17.89 (PMC13618340; doi:10.3762/bjnano.17.89)
Supplement: Supplementary file 1 [file Beilstein_J_Nanotechnol-17-1290-s001.pdf]

BEILSTEIN JOURNAL OF NANOTECHNOLOGY

Supporting Information

for

Design of a yeast-based bio-interface on an optical fiber SPR platform toward drug adsorption monitoring

Natsumi Kitaya, Daisuke Yamanaka, Kenji Morita, Ryota Naito, Hinako Suhara, Kento Suzuki, Hizuru Nakajima, Yukiko Moriiwa, Kazuhiro Morioka, Akio Yanagida and Atsushi Shoji

Beilstein J. Nanotechnol. **2026,** *17,* 1290–1301. doi:10.3762/bjnano.17.89

Additional figure and table

License and Terms: This is a supporting information file under the terms of the Creative Commons Attribution License (https://creativecommons.org/licenses/by/4.0). Please note that the reuse, redistribution and reproduction in particular requires that the author(s) and source are credited and that individual graphics may be subject to special legal provisions.
The license is subject to the *Beilstein Journal of Nanotechnology* terms and conditions: (https://www.beilstein-journals.org/bjnano/terms)

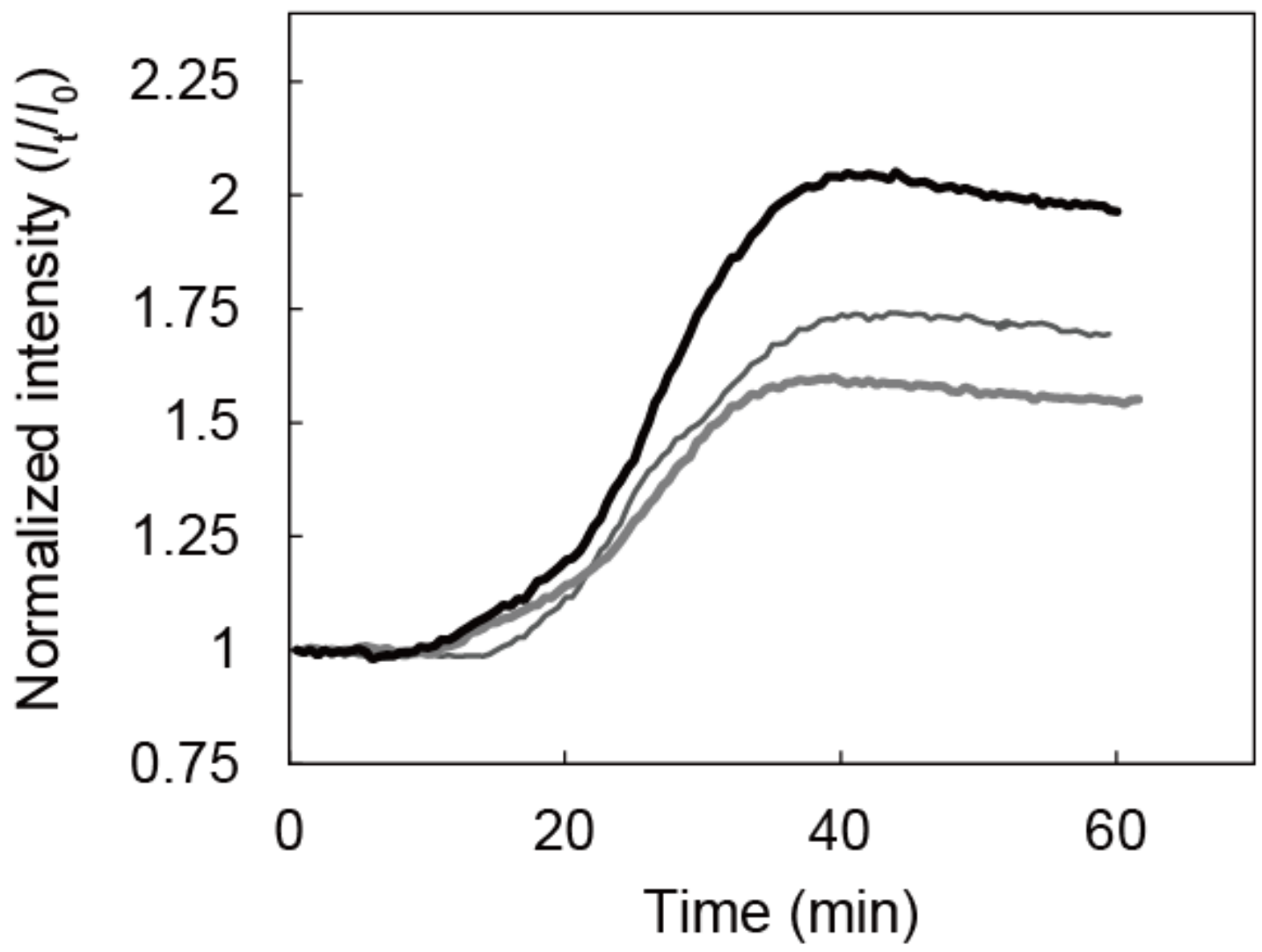

Figure S1: Change in reflected light intensity during the electroless nickel plating reaction. The reflected light intensity was measured at 55 nm. The profiles obtained using three different optical fibers are shown.

Table S1: Antimicrobial compound library.

Number	Compound Name
1	Rapamycin
2	Paclitaxel
3	Doxorubicin HCl
4	Bleomycin sulfate
5	Dacarbazine
6	Etoposide
7	Tamoxifen
8	Vincristine sulfate
9	Daptomycin
10	Doripenem Hydrate

11	Meropenem
12	Tigecycline
13	Linezolid
14	Cefaclor
15	Aztreonam
16	Cefdinir
17	Cefoperazone
18	Erythromycin
19	Chloramphenicol
20	Rifampin
21	Cefditoren Pivoxil
22	Sulfadiazine
23	Oxytetracycline
24	Thiotepa
25	Ethionamide
26	Bacitracin Zinc
27	Azithromycin
28	Sparfloxacin
29	Metronidazole
30	Sulfamethoxazole
31	Nystatin
32	Tebipenem Pivoxil
33	Honokiol
34	Nalidixic acid
35	Hordenine
36	Cephalomannine
37	10-Deacetylbaccatin-III

38	Lincomycin HCl
39	Mycophenolic acid
40	Novobiocin Sodium
41	Roxithromycin
42	Spectinomycin 2HCl
43	Ceftiofur HCl
44	Tetracycline HCl
45	Thiamphenicol
46	Geldanamycin
47	Amoxicillin
48	Daunorubicin HCl
49	Tazobactam
50	Nadifloxacin
51	Tylosin tartrate
52	Carbenicillin disodium
53	Ticarcillin sodium
54	Kitasamycin
55	Cefonicid sodium
56	Sulfachloropyridazine
57	Fusidine
58	Retapamulin
59	Erythromycin Ethylsuccinate
60	Spiramycin
61	Clofazimine
62	Tilmicosin
63	Ampicillin Trihydrate
64	Penicillin G Sodium

65	Doxycycline Hyclate
66	Mezlocillin Sodium
67	Dirithromycin
68	Piperacillin Sodium
69	Demeclocycline HCl
70	Cephapirin Sodium
71	Vinblastine sulfate
72	Cefotaxime sodium
73	Nitrofurantoin
74	Succinylsulfathiazole
75	Cephalothin sodium
76	Cefazolin Sodium
77	Cefixime
78	Dapson
79	Tedizolid Phosphate
80	Cefmenoxime hydrochloride
